# Unveiling laser diode “fossil” and the dynamic analysis for heliotropic growth of catastrophic optical damage in high power laser diodes

**DOI:** 10.1038/srep19011

**Published:** 2016-01-07

**Authors:** Qiang Zhang, Yihan Xiong, Haiyan An, Konstantin Boucke, Georg Treusch

**Affiliations:** 1 TRUMPF Photonics, Inc., 2601 US Route 130 South, Cranbury, NJ 08512, USA

## Abstract

Taking advantage of robust facet passivation, we unveil a laser “fossil” buried within a broad area laser diode (LD) cavity when the LD was damaged by applying a high current. For the first time, novel physical phenomena have been observed at these dramatically elevated energy densities within the nanoscale LD waveguide. The observation of the laser “fossil” is interpreted with different mechanisms, including: the origination of bulk catastrophic optical damage (COD) due to locally high energy densities, heliotropic COD growth, solid-liquid-gas phase transformations, strong longitudinal phonon cooling effect on the molten COD wave front, and the formation of patterns due to laser lateral modes. For the first time the COD propagation is analyzed temporally by an acoustic phonon bouncing model and the COD velocity is extrapolated to be exponentially decreasing from more than 800 μm/μs to a few μm/μs within a 20 μs time period as the energy density dissipates.

Laser diodes (LDs) are the most efficient electro-optical devices utilized to convert electrical energy to light with applications in industrial, civil, military, medical, communications and other extensive fields. Tremendously improved power, efficiency and beam quality have been achieved due to improved techniques of semiconductor laser design and epitaxial growth, packaging, facet passivation, and integration with novel optics. While it’s been long acknowledged that catastrophic optical mirror damage (COMD) is the dominant failure root cause for the high power GaAs-based LDs since its inception, fundamental solutions are still being explored to obtain COMD free LDs^1^,^2^,^3^,^4^,^5^,^6^,^7^. In this paper, we demonstrate a COMD level at the facet which is higher than the bulk damage threshold of LDs. This has been possible due to facet passivation by molecular beam epitaxy (MBE) under an ultra high vacuum (UHV) environment. LDs in 8-emitter laser arrays of 10mm array width and 4mm cavity length are passivated and used as test vehicles, and with this MBE passivation have shown a COMD current level higher than approximately 62 A per 90 μm-wide-emitter (100 us, 0.1% duty cycle) and have no apparent degradation after more than 8000 hour life time test (12 A per emitter, 0.5 Hz, 50% duty cycle). The life time test is still ongoing, demonstrating the high performance and long term robustness of passivated LDs. The development focus for the next generation LD will now focus on the elevation of the laser induced damage threshold (LIDT) of the bulk materials, namely the threshold of bulk catastrophic optical damage (COD), which has been observed in these MBE-passivated LD cavities.

The elevated COMD threshold leads to direct observations of novel phenomena within the semiconductor. These features include: the spatial origin of COD within the laser cavity, the consecutive phases of COD with exponentially decreasing velocities, the generation of longitudinal phonons, the phonon cooling effect of the molten COD wave front, the evolution of lateral laser modes of decreasing order, the redistribution of lateral modes at the interfaces, and the diffraction patterns indicating microscopic structures. A comprehensive interpretation is proposed for these observations and the dynamics of the COD growth is interpreted via a phonon bouncing model.

Laser induced phase transformations have been extensively studied for almost 50 years since the first report of its observation where the laser induced irreversible change was interpreted as a result of the intrinsic or extrinsic thermo-physical and metallurgical properties of the materials^8^,^9^,^10^,^11^,^12^. The theoretically calculated threshold fluence is only a logarithmic function of the electron density and the experimental damage threshold varies greatly with the material preparations and qualities, and the irradiation conditions^11^,^13^,^14^. It’s reported that the energy fluence threshold for permanent damage (F_th_) varies from 0.1 to 1.5 J/cm^2^,^7^,^8^,^12^,^13^,^14^. Our devices survive under a high energy fluence up to 1.76 J/cm^2^, probably resulting from our high-quality epitaxially grown semiconductor and the incorporation of Al, whose reported F_th_ = 1.2 J/cm^2^ irradiated with 620 nm laser. The laser-matter interaction physics within a confined transparent region is fundamentally different at high energy intensity (≥F_th_) from that at low energy intensity (<F_th_). There are distinct regimes of behaviors for different fluences: lattice heat (<0.5 F_th_), lattice disordering (0.6–0.8 F_th_), and a semiconductor to metal transition (>0.8 F_th_)^9^,^10^,^15^. For high fluences, the laser induced permanent changes occur due to a fast transition from semiconducting to metallic behavior, indicating the known non-thermal melting of the material. While for lower fluences, changes are reversible and lattice disordering occurs without the semiconductor-to-metal transition associated with melting.

The prevailing view of the intrinsic bulk degradation mechanism is the high carrier density induced lattice instability, which is conventionally considered only attainable for ultrafast lasers^9^. For single quantum well (QW) semiconductor lasers, it’s possible to attain high carrier densities which are enough to disorder the crystal, break bonds and ionize atoms, leading to the covalent crystal lattice instabilities^9^,^15^. In addition, an intense laser beam is further strongly self-focused by the thermal lensing effect in LD’s and the focused interaction zone is tightly confined within a region less than 1 μm^3^, leading to a locally higher energy density which can rapidly liquefy and even sublimate the solid. The phase-transformed solid can break the confinement of the surrounding lattice^16^.

Resembling an explosion of an energetic bomb of comparable energy density, the origin of the bulk COD causes the generation of shock-wave-like longitudinal phonons, the compression and decompression in the cold solid, and the expansion of the restricted material. The boundary velocities of the explosion can be in the order of 10^3^ m/s within a sub nanosecond^15^,^17^. The gas-phase formation of the components may also occur, leading to much faster diffusion velocities due to the high temperature and high elemental vapor pressure^15^. Longitudinal phonons generated due to the sudden energy burst propagate into the surrounding relatively cold semiconductor at different velocities according to anisotropic elastic moduli in the semiconductor. Phonon propagation results in the periodic density and stoichiometric variation in three-dimensions in the mono-crystalline semiconductor. This is further pronounced by the longitudinal bouncing back and forth between the facet and the COD liquid-solid interface along the laser cavity. Transverse phonon propagation might be unlikely occur due to the lack of shear modulus for the molten crystal fluid.

The bulk COD wave front according to the classical picture of melting and resolidification propagates inhomogeneously via the phase transformation boundary^15^. The bulk COD wave front propagates at a very high velocity along laser cavity at the very beginning, which might be related to the material properties, and then decelerates rapidly. For the COD originating near the AR facet, the interaction between the COD wave front and the longitudinal phonons reflected from the AR can be neglected because the phonons are scattered and minimally affects the COD wave front when it catches up the decelerated COD wave front. In contrast, the longitudinal phonons propagating in the unaffected laser cavity are reflected from HR facet towards the COD wave front, partially transmit through, and then reflect back to the HR facet. The energy density of COD wave front increases at the beginning of the interaction due to the energy addition from the phonons and then quickly drops with energy transfer to the partially transmitted and reflected phonons, which have extracted energy from the COD, leading to a pulse-like compression of the molten COD.

It should be noted the semiconductor could further be heated up via stimulated Brillouin scattering (SBS), a strong photon-phonon interaction due to the high intense laser induced non-linearity when the intensity of the light field itself affects the propagating medium^18^,^19^,^20^. Further tests are needed to verify such SBS effects within a LD. It has recently been reported of the giant enhancement of SBS in the order of 10^5^ times in the waveguide system with nanoscale engineered geometry^20^,^21^. The enhancement may be further boosted due to the decreased threshold of optical intensity for broad-band pump lasers^22^.

## Experiments

**Figure 1:**
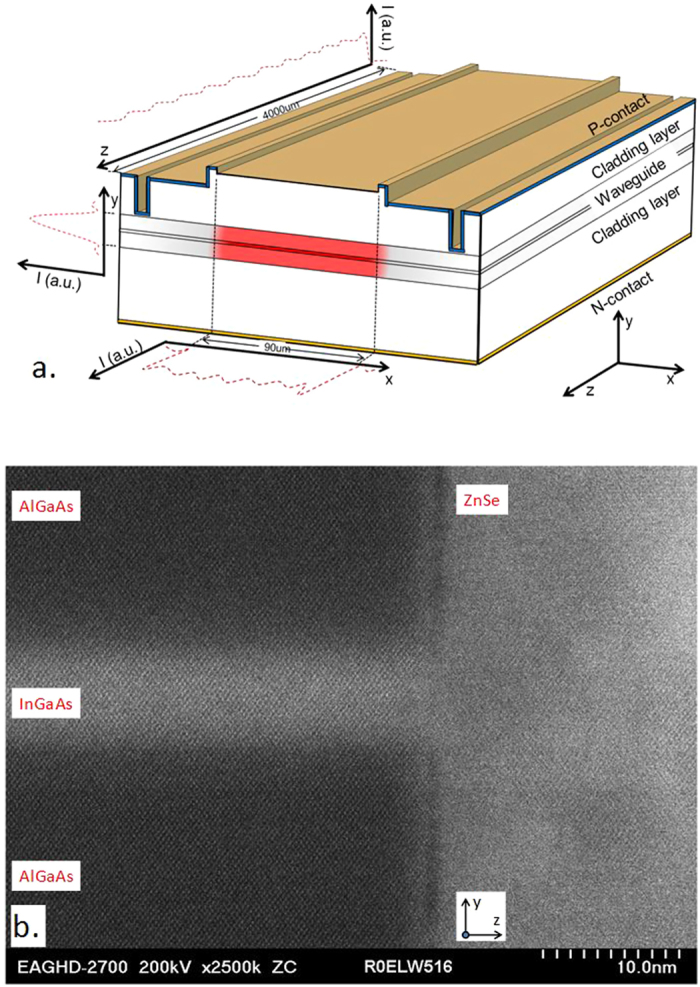
Schematic of a broad area laser diode and XSTEM of the 3D epitaxial growth. (**a**) shows the electro-optical conversion layers grown in y-axis direction and the facet passivation layer grown in z-axis direction. The cavity length of LD examples is 4000 μm and the p-contact window width is 90 μm. When a current is applied from p-contact window through LD to n-contact, LD is designed to emit near infrared light in a fundamental mode in y-axis and multiple modes in x-axis and z-axis. (**b**) shows the XSTEM image of the 3D epitaxially grown semiconductors. The AlGaAs waveguide layers and InGaAs quantum well layer are grown by MOCVD in y-axis direction and ZnSe passivation layer is grown by MBE in z-axis direction. The 3D grown epitaxial semiconductors show clearly resolved lattice sites and are defect free.

The broad-area lasers are grown on a (100) +/− 0.1° Si-doped n-GaAs substrate by metal-organic chemical vapor deposition (MOCVD). A schematic drawing of the broad area laser diode is shown in Fig. 1-a. The active region, consisting of an InGaAs single QW confined in an AlGaAs waveguide and cladding layers, is designed to emit light at around 938 nm of single mode in the y-axis and multiple modes in the x-axis and z-axis. The power conversion efficiency is approximately 65% at the operational current of 9 A per emitter^2^. The front and back facets are passivated with lattice-matched zinc selenide (ZnSe) by MBE under a UHV environment followed by *in-situ* coating with dielectric layers by ion beam sputtering asymmetrically, leading to a 2% anti-reflective (AR) and a 97% high-reflective (HR) coating, respectively. The laser is mounted p-side down on a coefficient of thermal expansion matched sub-mount with AuSn hard solder. This sub-mount is then soldered to a Cu-heatsink.

The current source used for the experiment provides a maximum current of 500 A, pulse width of 100 μs and duty cycle of 0.1%. An increasing current was applied with 20 A steps and the laser was operated for 1 minute during each step or until laser failure. The results were obtained based on 88 emitters from 11 laser arrays. The emitters can be regarded as individual emitters in our time scale which is too short to allow any lateral thermal crosstalk, assuming the homogeneous current distribution to each emitter^23^.

The epitaxial regions of failed emitters were then inspected by removing bond wires, n-side lapping and polishing, and chemical etching. Ammonium hydroxide-hydrogen peroxide (NH_4_OH-H_2_O_2_) mixture was chosen for the selective etching of the substrate with a selective ratio of around 800 for GaAs-to-Al_0.6_Ga_0.4_As in (100) crystalline orientation. Higher values of selectivity are expected for higher Al compositions^24^,^25^,^26^.

The waveguide and facet were inspected by cross-sectional scanning transmission electron microscope (XSTEM) and the epitaxial layers were inspected by a Nomarski Differential Interference-Contrast Microscope, Secondary Ion Mass Spectrometry (SIMS), Tencor P-10 Profiler, and a Bruker Dimension Atomic Force Microscope (AFM).

## Results

The novel phenomena to be described below are made possible due to the robust passivation layers, which are epitaxially grown on the facets and characterized by XSTEM, as shown in Fig. 1-b. The lattice sites are clearly resolved for the electron-optical conversion layers (AlGaAs/InGaAs/AlGaAs) grown along y-axis by MOCVD and the ZnSe passivation laser grown along z-axis by MBE, indicating almost defect free 3D epitaxial growth.

**Figure 2:**
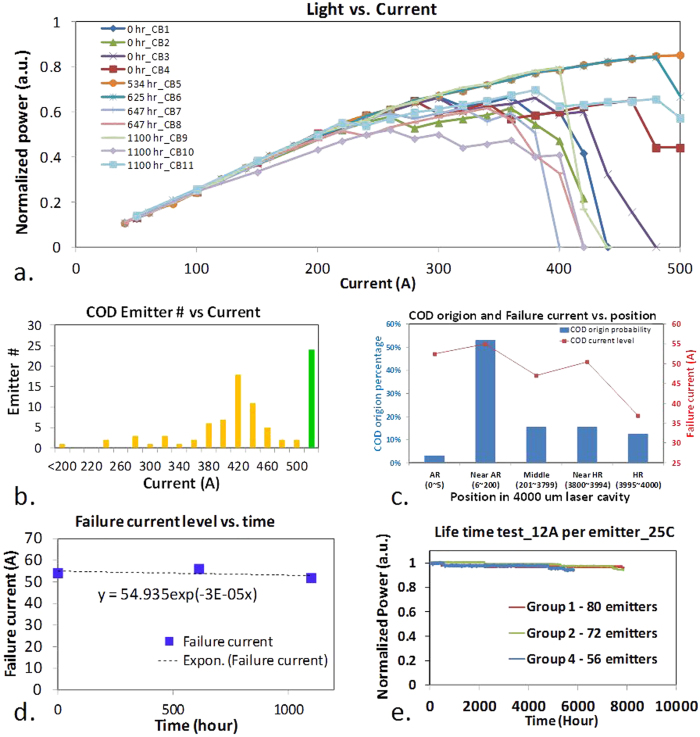
Performance and reliability of LDs. As shown in the plot of light vs. current in (**a**), most emitter failed at around 420 A per laser array (~53 A/emitter), while some emitters survive at 500 A (62 A/emitter), which is the limit of our current source. (**b**) shows the distribution of COD numbers at different COD current is shown in inset. (**c**) shows the distribution of COD origin and the current level at which the emitters failed. The failures mostly occurred near AR and nearly evenly occurred in the middle, near and on the HR. The highest average COD current level (55 A/emitter) occurs when the COD originated near AR. The failure current level of emitters shows little degradation within approximately 1100 hours of aging test as shown in (**d**), and lasers show no apparent degradation for approximately 6500 hours of aging test as shown in (**e**). The aging test is under 12 A per emitter, 0.5 Hz, 50% duty cycle and is still ongoing.

The plot of light-current (L-I) for 8-emitter laser arrays is shown in Fig. 2-a. Apart from lasers that did not fail, most lasers failed at around 420 A as shown in Fig. 2-b, corresponding to a current density of 15 kA/cm^2^ based on a 90 μm × 3960 μm (width × length) contact opening area. As mentioned previously each laser array was tested with 20 A steps until the laser failure or the limit of the current source 500 A. Failed lasers were thereafter inspected by a Nomarski optical microscope, but there were no apparent signs of defects on AR, HR or the n-side of the lasers. Thus, COMD could be excluded from failure root causes. That means the laser induced damage threshold of facets is more than 0.7 A/μm. 32 emitters of the failed ones were inspected after substrate removal. A total of 84% bulk-COD originated within the laser cavity. The COD origin distribution shows approximately 53% near the AR facet, and the highest average COD current level (55 A/emitter) occurred if the COD originated near AR as shown in Fig. 2-c. The failure current level of each emitter shows little degradation within approximately 1100 hours under aging test, as shown in Fig. 2-d. In addition, lasers have no apparent degradation after approximately 8000 hour aging test (12 A per emitter, 0.5 Hz, 50% duty cycle), and the aging test is still ongoing, as shown in Fig. 2-e. It has been reported that higher bulk-COD current levels have been observed for shorter pulses and remains almost constant for 2 ms up to continuous wave (cw) irradiation. Damage threshold achieved in this work (~0.7 A/μm) is significantly higher than previously reported levels of 0.05 A/μm for 100 μs pulse laser^5^ and 285 mw/μm for cw laser^27^.

**Figure 3:**
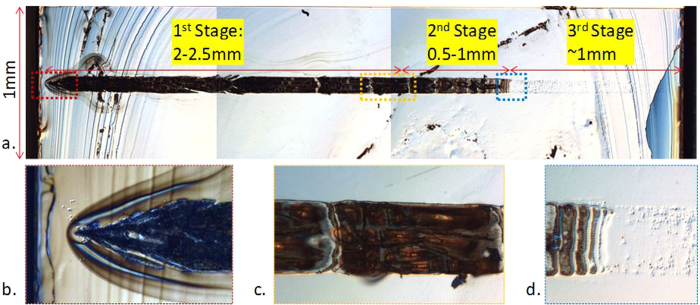
A typical top view of a laser “fossil” (E1). The image was taken by the optical microscope. There are 3 distinctive stages of the pattern shown in (**a**) from the COD origin: In the 1^st^ stage from COD origin shown in (**b**) to approximately 2 to 2.5 mm shown in (**c**), the profile of the COD shell is rough and show plateaus of 5 to 20 μm in y-axis; in the 2^nd^ stage from (**c**) to the tail of COD shown in (**d**), a smooth y-z profile with clear periodical structures along x-axis is shown for approximately 0.5 to 1 mm. The profile is around 0.5 μm in y-axis and described in details in Figs 4 and 5; in the 3^rd^ stage from (**d**) to the end of the laser cavity, a flat profile with small hillocks and dents starts from the tail of the COD pattern and fades away until the facet shown in (**d**). The 3^rd^ stage is not apparently observed for all failed emitters, probably due to the different levels of crystalline deformation and sample preparation.

After removing the substrate, emitters that survived after applying 500 A current do not show apparent defects in the epitaxial region. COD patterns are only observed in active regions only for failed emitters, as shown in Figs 3 and 5, [Fig f5] for three typical emitters with COD near AR. Chirped longitudinal periodic structures are only clearly observed in the epitaxial regions of emitters damaged during high-current L-I testing, but not clearly observed for emitters damaged during aging tests under lower current conditions (~12 A per emitter). This indicates that the laser finger print “fossil” occurs only under extremely high current density and power density in the laser cavity, which is only approachable due to the robust facets with significantly elevated COMD threshold.

## Discussion

Literature reports that etch rates vary dramatically with semiconductor composition, material quality including amorphous/crystalline quality, crystal orientation, and doping concentration, etc.^24^,^25^,^26^. This helps to clarify the root causes for the observed damage patterns observed within the laser diode waveguide that are revealed by the selective etching process. The damage initiation point may introduce phase changes, deteriorations or deformations of different levels in each semiconductor at different laser intensities. Deteriorated and un-deteriorated compound semiconductor regions are etched away at different etching rates due to anisotropic etching, so that it is readily attainable to unveil the laser “fossil” within the laser cavity when the laser is damaged, as shown in Figs 3 and 5, [Fig f5] of three typical emitters named as E1, E7 and E8, respectively.

At the initiation of damage the energy density is so high that all semiconductors near the COD origin melt and transform from semiconducting solid to the metallic liquid phase, leading to a large increase in light absorption. Note, that the COD origin is not necessary at or near the QW region, where it is mostly suspected to be vulnerable to the laser damage^5^. The new melt near the liquid-solid boundary is expected to have the same composition as the surrounding crystalline materials initially, and then the composition of melt is expected to be averaged in a larger scale or in the ensemble of the molten semiconductor, depending on the elemental diffusion velocities in the semiconductor in liquid phase. The waveguide and cladding layers consist of Al_x_Ga_1–x_As, where x varies from 0 up to 0.6 with an average value of 30% Al atomic concentration for the entire epitaxial grown structures.

As the melt starts to solidify, from the boundaries where the temperature drops faster than the inner parts of the melt, the Al-rich components will firstly condensate on the boundary between the melt-solid interfaces based on the pseudo-binary phase diagram for GaAs-AlAs^28^. AlGaAs of higher aluminum composition, which is more chemically robust to the etching solutions, is expected near the shell of the COD pattern. This will form the observed profile after etching of the damage pattern. Higher aluminum composition was also observed in the COD patterns with SIMS, indicating aluminum diffusion in the adjacent regions during the COD events (Supplementary Fig. S1).

In addition, the explosion and expansion which is confined within semiconductor, indicated by the elevated bumps above the active regions, results in a high hydrostatic pressure which depresses the melting point of GaAs^29^. The etch rate of (110) GaAs is around 50% higher than that of (100) GaAs, resulting from the very different surface activities on the semiconductor-solution of Ga rich and As rich faces, that is, the As-rich face is more reactive than the Ga-rich face. It is believed that a combination of all these factors lead to the selective chemical etching of the observed damage patterns.

**Figure 4:**
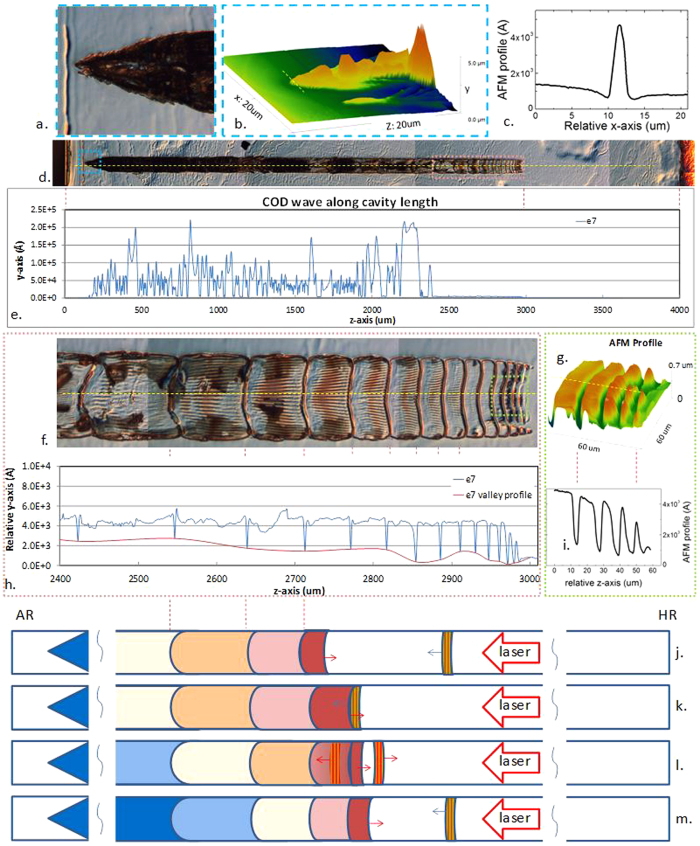
Phonon bouncing model to interpret bulk-COD node formation. A typical top view of COD pattern (E7) is shown in (**d**). The left side is AR and the right side is HR. The COD pattern profile along the cavity indicated by a yellow dashed line is measured by a profiler and shown in (**e**). The initial stage of the COD marked a blue dashed rectangular is zoomed and inspected by optical microscope in (**a**), and by AFM in (**b**). The AFM profile along the yellow dashed line in (**b**) is plotted in (**c**), showing a less than 3 μm COD origin width. The COD tail pattern marked by a pink dashed rectangular in (**d**) is zoomed in (**f**). Its profile along the cavity indicated by a yellow dashed line is measured by a profiler and shown in (**h**). The end of the tail is inspected by AFM in (**g**) and the AFM profile along the yellow dashed line is shown in (**i**). The model is illustrated in (**j–m**) to simulate the formation of a node in the laser “fossil”.

**Figure 5 f5:**
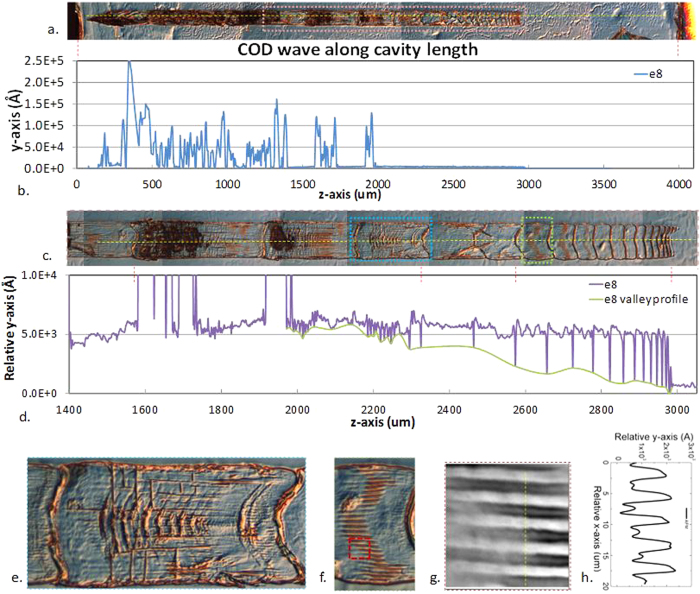
Diffraction patterns and lateral fine structures in a laser. A typical laser “fossil” (E8) is shown in (**a**) and the profile along the yellow dashed line is shown in (**b**). The area inside the pink rectangular is zoomed in (**c**) and its profile along its yellow dashed line is shown in (**d**). Regions marked by the blue and green rectangles in (**c**) are zoomed in (**e,f**). A diffraction pattern along the z-axis is shown in (**e**) and a fine periodical structure with shoulders along x-axis is shown in (**f**). The area inside the red rectangle is inspected by AFM shown in (**g**) and the profile along the yellow dashed line is shown in (**h**), showing an approximate 4 μm periodicity with lobes. Note that the periodicity increases along with the COD propagation in the rage of ~2.5 μm to ~4 μm.

Figure 3-a–d show a typical COD pattern where there are 3 distinctive stages from the COD origin: The first stage is from COD origin to approximately 2 to 2.5 mm, and a rough y-z profile is observed of 5–20 μm in y-axis [Fig. 4-e]. In the 2^nd^ stage, a smoother y-z profile with clear periodical structures along x-axis is shown for approximately 0.5 to 1 mm. The profile height is around 0.5 μm in y-axis [Figs 4 and 5]. Note, that the smooth profile can be also observed in the end of the 1^st^ stage as shown in Fig. 5. In the 3^rd^ stage, a relative flat profile with small hillocks and dents is observed from the end of the tail of the COD periodic pattern and fades away until the rear facet is reached. The lateral width in the x-direction is slightly narrower with COD propagation in the 3^rd^ stage than that in the first two stages. Note that the 3^rd^ stage is not apparently observed for all failed emitters, probably due to the different levels of crystalline deformation and sample preparation.

The three distinctive stages of the COD damage profile indicate three different deformation mechanisms. In the 1^st^ stage, most COD is originated from a point near the front facet and grows heliotropically (“grows towards the light source”), that is, the COD propagation is very directional facing the optical energy reservoir in z-axis, resulting from the energy feed from laser via free electron absorption in the metallurgic COD. COD can also originate from a few points nearby, probably due to mode re-distribution after the initial COD origination^5^, as shown in Fig. 4-a,b, inspected by optical microscope and AFM, respectively. The COD origin is less than approximately 3 μm in x-axis and greater than 0.3 um in the y-axis, as shown in Fig. 4-c. The rough profile in the 1^st^ stage indicates there are violent explosions, as shown in Figs 4-e and 5-e, corresponding to the ultra-high temperature induced solid-liquid-gas phase transformations. Mixtures of non-stoichiometric semiconductors and possible elemental gasses expand up to tens of micrometers, In the 2^nd^ stage, a smooth x-z profile appears, indicating a smooth solid-liquid transformation corresponding to a lower COD temperature, as shown in Figs 4-f–I and 5-f,g. Clear chirped periodical nodes are observed in the first two stages, while only a trace of deformation instead of the re-solidified COD shell is observed in the 3^rd^ stage, as shown in Fig. 3-d. This indicates a deformation mechanism other than a phase transformation, possibly due to the mono-crystalline orientation change from (100) orientation in y-axis to a different orientation.

In the 1^st^ stage, the laser induced COD origin expands fast in the z-axis and slow in the transverse axes, as indicated in Fig. 4-b of the 3D AFM image. The COD origin angles are the indication of the inhomogeneous COD expansion and found between 22° and 38°; which is higher than the reported values at lower COD levels^5^. The varied angles indicate that at the beginning, the COD moves about 1.3 ~ 2.5 times in z-axis longer than that in x-axis. The growth anisotropy in the longitudinal and transverse directions results from the different thermal mechanisms. A non-equilibrium thermal process exists in z-axis because the COD absorbs energy when it moves facing the light radiation while there is little energy feed in transverse directions, leading to the heliotropic growth mode. Note that the anisotropic elastic properties in semiconductor are not taken into account for the COD growth difference due to the inelastic melting process of the solid-liquid phase transformation. In the 1^st^ stage, spiky profiles with elevated plateaus and diffraction patterns are observed, as shown in Figs 3 and 5, [Fig f5]. The stochastic spiky profiles could be related with gas phase transformation and short energy spikes resulting from laser interference, which could be considerably greater than the mean energy of the laser^30^. The energy spikes lead to Al explosive diffusion and the spiky shell formation is due to III-V non-stoichiometric composition distribution^31^.

Accompanying the COD propagation, energy dissipates through the COD boundaries and the environment in all directions. In x-axis and y-axis, energy loss is dominant and the COD propagation in these 2 dimensions rapidly decreases. At the beginning of the COD, the temperature is so high that the COD can melt GaAs beyond the AlGaAs waveguide for more than a few micrometers in y-axis. After it travels around 150 μm along the laser cavity, the COD width in x-direction increases to 90 ~ 100 μm and remains, slightly wider than the designed contact opening width of 90 μm, which could be related to current spreading. After it travels around 2 ~ 2.5 mm, the average COD width slightly decreases to around 90 μm and remains this width for a distance of 0.5 ~ 1 mm until the COD stops propagating, as shown in Figs 4-f–i and 5-c–h. In this 2^nd^ stage, the energy density is decreasing but is still above the damage threshold and is still high enough to trigger an irreversible solid-liquid phase transformation^8^. Convex and concave COD wave fronts in z-direction and sharp edge boundaries in y-axis and x-axis are observed. The COD patterns show clearly nodes with chirped periods which are longer near the COD origin and become shorter with the increasing distances from the COD origins, as shown in the profile plots in Figs 4-d–h and 5-a–d. There are fine lateral periodical structures between nodes whose periods decrease gradually with increasing node number, denoting the COD origin as the initial node.

An acoustic phonon bouncing model is proposed to interpret the COD longitudinal dynamics in Fig. 4-j–m. A longitudinal phonon generated at the COD origin bounces back from the HR facet with nearly 100% energy conservation from the edge because the negligible energy loss via the solid-air interface, and propagates towards the COD wave front, as shown in Fig. 4-j. The phonon partially reflects and transmits via the COD solid-liquid interface, as shown in Fig. 4-k,l. The reflected phonon then bounces back from the facet again while within a shorter cavity, as shown in Fig. 4-m. When the longitudinal phonon collides with the COD wave front, the energy density rises due to phonon energy inclusion locally along the COD boundaries, resulting in the rise of the profile before the valleys, as shown in Figs 4-h and 5-d. The cold longitudinal phonon extracts heat from COD and carries away the energy by partially transmitting and rebounding back in the cavity, leading to a sudden drop of energy density and the shrinkages of the COD wave front, and thereby the nodes are imprinted where the interaction occurs between the COD and the longitudinal phonon. The COD wave front velocity decreases with decreasing temperature, which leads to the chirped patterns and can be used to analyze the COD dynamics. The phonon bouncing model is consistent with the observed diffraction patterns. The molten semiconductor behind the COD wave front cools down from the side to the center leaving a narrow channel in the middle so that the transmitted acoustic phonon, which is also the sound wave, diffracts through it, as shown in Fig. 5-e.

**Figure 6:**
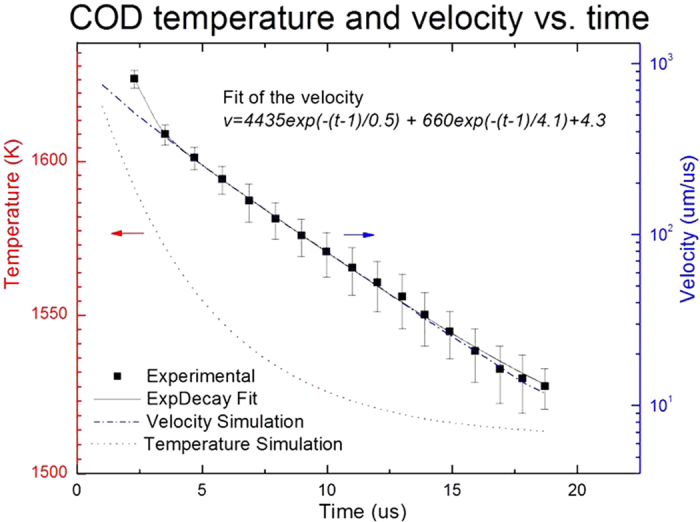
Exponentially decayed bulk-COD velocity. Based on all emitters with COD near AR and the bouncing model, the average velocity (black square) is calculated based on the distance between nodes by taking a constant phonon velocity of 5238 μm/μs and plotted as a function of time. By fitting the curve, COD wave front velocity decreases via 2 distinctive exponential decay times: a fast decay time ~0.5 us at the beginning and a slower decay time ~4.1 us, indicating different mechanisms. The slower decay mechanism is numerical simulated showing that the temperature (red dotted line) is more than 150 K higher than the melting temperature 1511 K at the beginning and the velocity (blue dashed line) exponentially decreases with time.

The dynamics of the COD wave front takes place in a single pulse and is analyzed based on the proposed phonon bouncing model and the COD originating near AR, where the highest average COD current level occurred, and the influence of the initial location of the COD on the form of the phonon propagation will be studied in more details in future papers. In the first two stages, the velocity of the molten COD solid-liquid wave front is a function of its temperature^9^. The average velocity is calculated based on the distance between consecutive nodes by taking a constant phonon velocity 5238 μm/μs^31^. The average velocity within two consecutive nodes can be plotted as a function of time, as shown in Fig. 6. By fitting the velocity-time curves, an equation is obtained:


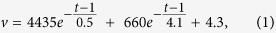


where 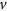 is the COD velocity and 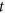 is time. There are 2 different decay times: a fast decay time ~0.5 μs and a slow decay time ~4.1 μs, corresponding to different COD propagation mechanisms. A numerical simulation shows the initial temperature can be more than 1650 K and the initial velocity of the COD wave front could be much higher than the average velocity, ~800 μm/μs, within the first node. By a rough estimate, it takes ~1 μs for a phonon bouncing between COD wave front and facet, and COD velocity is ~200 μm/μs at ~3 μs after the COD origination, which is consistent with the reported values from 30 m/s to 190 m/s during 300 ns laser induced melting in LD’s at a lower current density^32^, and from 250 m/s to 500 m/s for the liquid-solid interface velocity during picosecond laser induced melting of GaAs^15^. The profile of the node depth also shows a chirped periodical fluctuation, as shown in red profile in Fig. 4-h and in green profile in

Figure 5-d, respectively, indicating an interaction with fluctuated energies between the COD wave front and the phonon. In the region around the COD tail, the velocity of the COD wave front is affected more by other complex mechanisms, such as the heat redistribution due to SBS, the latent heat effect when the liquid-solid interface temperature is around the melting temperature and band gap shrinkage effect for the semiconductor at elevated temperatures. The COD wave front propagation fades away when the temperature of COD wave front is near the melting temperature and there is a temporary equilibrium of the energy feed and heat dissipation.

Light induced COD wave propagation also shows periodical patterns with lobes laterally, as shown in Fig. 5-f–h, resulting from the manipulation by the spatial distribution of light intensity, namely laser lateral modes^33^,^34^. The period increases from around 2.5 μm to 4 μm with the COD propagation, indicating the decreasing number of the optical modes, which is consistent with the decreasing laser intensity due to the reduction of cavity length during its propagation. The lateral modes between consecutive nodes are stable, while the much blurred periodic patterns are observed near the nodes, which can be related to the interface disturbance.

Laser lateral mode number is closely related to the thermally-induced indices of refraction. The thermal dependencies of the indices of refraction of GaAs and AlAs for wavelengths near 1 μm were reported to be (2.67 ± 0.07) × 10^−4^/°C and (1.43 ± 0.07) × 10^−4^/°C, respectively^35^. The change of thermal induced refractive index can be more than ~3 × 10^−2^, when the temperature difference is more than 110 K. Note that the periodical structures may be also affected by the laser interferences, laser filamentation and spatial hole burning effect, which results from the spatially non-unifomity of the photon density and the carrier distribution.

In summary, we have reported the elevated COMD threshold and the observations of novel physical phenomena within the semiconductor LD following bulk-COD. A phonon bouncing model has been proposed to analyze the dynamics of the COD propagation and a comprehensive interpretation has been proposed for the other observations, such as: the origin of COD, the evolution of the consecutive phases of COD with exponentially decreasing velocities and with decreasing power density levels, the formation of lateral laser modes of decreasing orders, the redistribution of lateral modes at the interfaces, and the diffraction patterns indicating microscopic structures. The finger print of laser modes that can be solidified in a high power laser may open new avenues for the study and development of higher power semiconductor electro-optic devices.

## Methods

### Device fabrication

Electro-optical conversion layers of the device were designed and optimized for a low internal loss and epitaxially grown by MOCVD (Aixtron 1615). Wafers with metal contacts are cleaved to 1 × 4 mm^2^ laser bars, and the facets of the bars are processed based on an all in-vacuum 4-step procedure, which includes facet outgassing, lower kinetic atomic hydrogen cleaning, defect free epitaxial passivation by MBE (Riber 412) and dielectric coating by ion beam sputtering deposition (Oxford Ionfab 300Plus). Laser bars are then mounted on heat sinks and bounded with gold wires.

### Measurement and experimental set-up

The light-current measurement were carried out with an Amtron CM392 current source, whose maximum current is 500 A, current rise up time is less than 1 μs, pulse width is 100 μs and duty cycle is 0.1%. The near field images taken by a CCD camera and the normalized light peak intensities were measured by an integrating sphere and a photodiode. An increasing current was applied with 20 A steps and the laser was operated for 1 minute during each step until the laser failure. The results were obtained based on 88 emitters from 11 laser arrays. The emitters can be regarded as individual emitters due to low fill factor 10% and short pulse duration 100 μs, where lateral thermal crosstalk is not likely to happen.

### Sample preparation

The epitaxial regions of 32 failed emitters were examined by removing wire-bonds, n-side lapping and polishing, and selective chemical etching. The thickness of the sample was thinned to 12.5 μm, which was monitored by micrometer and measured by step profiler. The selective etching solution used is an ammonium hydroxide-hydrogen peroxide (NH_4_OH-H_2_O_2_) mixture with 50% concentrated hydrogen peroxide (H_2_O_2_) and 30% concentrated ammonium hydroxide (NH_4_OH). The composition of the solution given by the volume ratio r = V_H_/V_A_ is the parameter for varying the etch rate and insuring the selectivity of GaAs to AlGaAs, which is larger than two orders of magnitude during the etching process. Higher value of selectivity is expected for higher Al composition. Agitation is applied during the etching process. After etching, the sample was taken out of the etching solution, rinsed by methanol and dried by nitrogen gas.

### Model and numerical simulations

The velocity of the molten COD solid-liquid wave front is a function of its temperature^9^. The energy transportation during the COD propagation


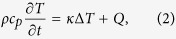


where 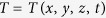 is the temperature, which is calculated by means of the heat equation and determines the excursion of the molten semiconductor profile; 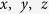 are space coordinates and 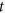 is time; 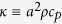 is the thermal conductivity and 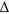 is the Laplace operator, 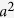 is the thermal diffusion coefficient, and 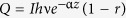 is the power of heat sources associated with laser irradiation absorption and material properties.

With the parameters in table 1, the COD wave front velocity can be numerically simulated^9^,^36^,^11^,


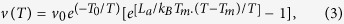


where 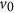 and 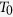 are the parameters determined by microscopical material properties and 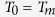 is applied in the simulation. Due to the strong metallic absorption, the light is considered to be entirely absorbed at the COD wave front, 
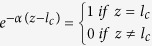
. Under a heat equilibrium condition at the end of the COD propagation 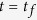, 
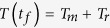
, where 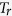 is the temperature offset with the melting temperature to sustain the COD propagation; the heat loss 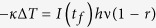. The temperature can be reversely derived from the heat equilibrium condition,


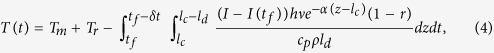


where 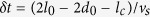 is the time interval between the consecutive COD-phonon interactions before the end of the COD propagation; 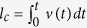 is the COD propagation distance;
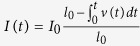
 is the active cavity length dependant photon flux density and 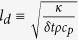 is the COD thermal diffusion length.

At the beginning of the COD, based on the observation of the width of the COD origin in x-axis, assuming laser irradiation is largely converged to a region 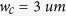 in x-axis with depth of focus of the beam 
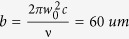
, and the photon flux 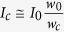. It takes less than 1 μs to melt the focused area, based on a one-dimensional solution of the heat equation corresponding to the maximum heating^11^,^36^.


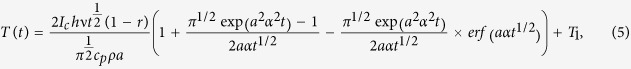


where 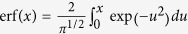; 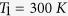 is the heat sink temperature. The assumed size and time for COD origin is comparable with the reported primary damage of 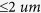 width and sub-microsecond time scale^17^.

## Additional Information

**How to cite this article**: Zhang, Q. *et al.* Unveiling laser diode “fossil” and the dynamic analysis for heliotropic growth of catastrophic optical damage in high power laser diodes. *Sci. Rep.* **6**, 19011; doi: 10.1038/srep19011 (2016).

## Supplementary Material

Supplementary Figure S1

## Figures and Tables

**Table 1:** Physical parameters of GaAs.

Physical parameters	Symbol	Value	Reference
Longitudinal acoustic wave speed along [110] at 300 K	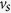	5.24 × 10^5^ cm s^−1^	31
Melting temperature (crystalline)	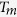	1.511 × 10^3^ K	11
Latent heat of fusion per GaAs molecule (crystalline-liquid)	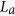	1.79 × 10^−19^ J/molecule	11
Density (crystalline at 1500 K)	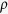	5.20 g cm^−3^	11
COD wave front Reflectivity (crystalline-liquid)	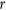	0.15	9,36
Thermal conductivity	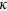	0.07 W cm^−1^ K^−1^	11
COD thermal diffusion length for bouncing time interval 1 × 10^−6^ s	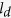	1.75 × 10^−4^ cm	11
Absorption coefficient at 825 K	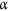	2 × 10^3^ cm^−1^	11
Specific heat (crystalline at 1500K)	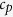	2.34 J K^−1^ cm^−3^	11
Material parameters	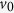	2.50 × 10^5^ cm s^−1^	31
Temperature offset	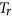	2.2 K	
Laser frequency	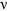	3.16 × 10^14^ cm	
Optical power	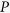	45 W	
Photon flux density for 45 W optical power at λ = 9.5 × 10^−5^ cm	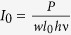	5.97 × 10^22^ cm^−2^ s^−1^	
Contact opening width	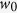	9 × 10^−3^ cm	
Laser cavity length	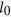	0.4 cm	
Boltzmann constant	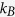	1.38 × 10^−23^ J K^−1^	
Planck’s constant	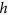	6.62 × 10^−34^ J s	
COD propagation time	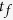	1.96 × 10^−5^ s^−1^	
Position of COD origin along z-axis	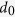	5 × 10^−3^ cm	
