# Supplementary material for: Unveiling laser diode “fossil” and the dynamic analysis for heliotropic growth of catastrophic optical damage in high power laser diodes
Source: Sci Rep. 2016 Jan 7;6:19011. doi: 10.1038/srep19011 (PMC4704051; doi:10.1038/srep19011)
Supplement: Supplementary Figure S1 [file srep19011-s1.pdf]

# Unveiling laser diode "fossil" and the dynamic analysis for heliotropic growth of catastrophic optical damage in high power laser diodes

**Qiang Zhang, Yihan Xiong, Haiyan An, Konstantin Boucke, Georg Treusch**

**TRUMPF Photonics Inc., 2601 U.S. Route 130 S, Cranbury, NJ 08512, USA**
**Correspondence and requests for materials should be addressed to Q. Z. (Email: ccnyqzhang@gmail.com, Tel: 1-609-925-8120, Fax: 1-609-409-7021)**

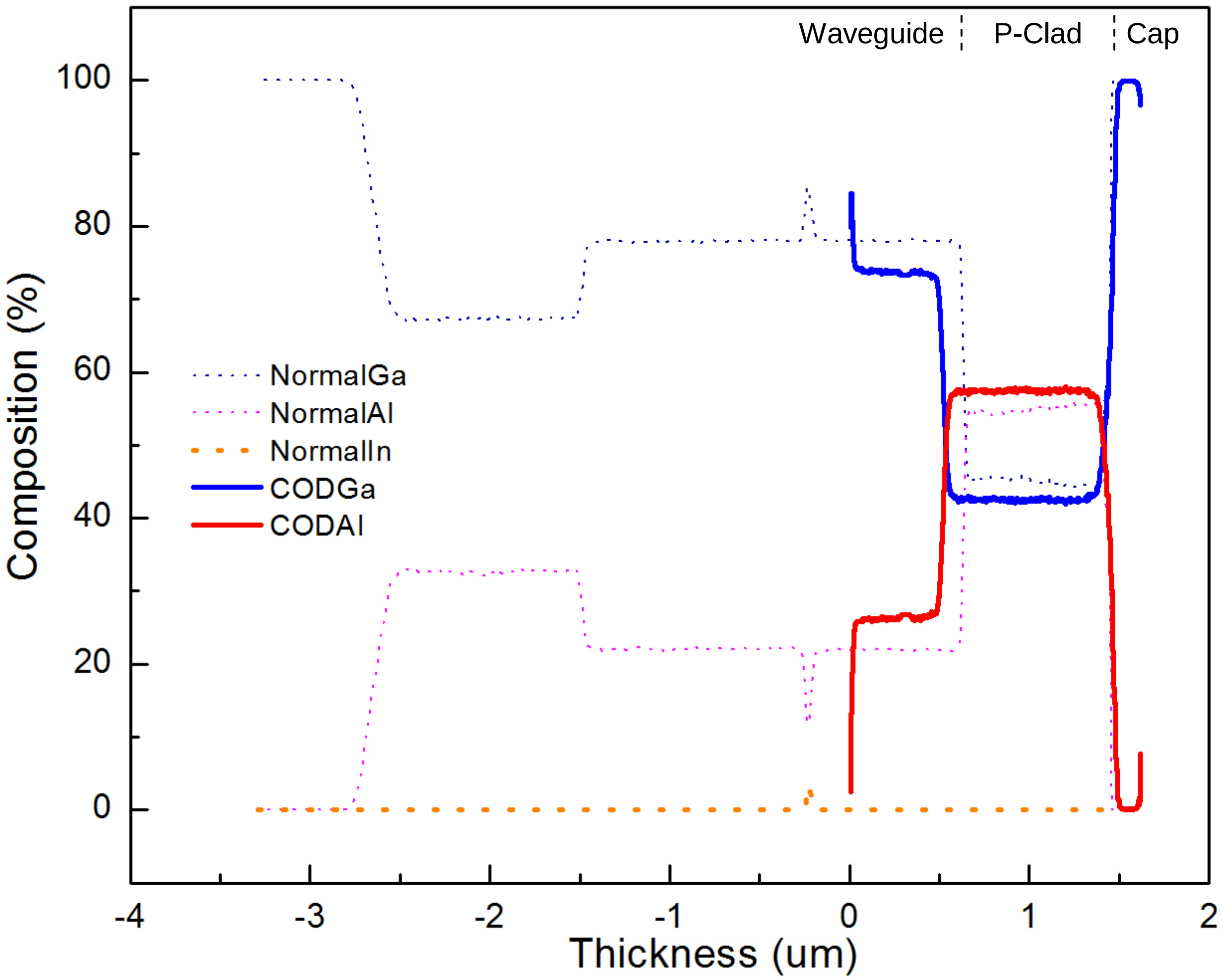


**Figure S1: The SIMS depth profiles in the COD pattern in Fig. 5-f.** The profiles of elemental Ga, Al and In of a normal device without a COD event are plotted for comparison. The concentration of In is not detected in the COD pattern. Clearly it is observed that the concentration of Al in the COD pattern is higher (~5%) than that in the normal device without a COD event, indicating aluminum diffusion in the adjacent regions during the COD events[1]. The segregation of Al-rich components may occur more likely on the boundary between the melt-solid interfaces, as observed by Hempel, M., *et al.*[2]. AlGaAs of higher aluminum composition, which is more chemically robust to the etching solutions, will form the observed profile, which is the shell of the COD pattern, after etching of the damage pattern. Note that the SIMS was performed from “Waveguide” to “Cap” for the depth profile in the COD pattern, while the sputtering direction is reversely from “Cap” to “Waveguide” in the normal device.


1. Frigeri, C., *et al*, Optical and structural analysis of degraded high power InGaAlAs/AlGaAs lasers. Mater. Sci. Eng. **B66**, 209-214 (1999).
2. Hempel, M., *et al.*, Microscopic Origins of Catastrophic Optical Damage in Diode Lasers. *IEEE J. Sel. Top. Quantum Electron.* **19** (4), 801 (2013).
